# Poor self-rated health predicts the incidence of functional disability in elderly community dwellers in Japan: a prospective cohort study

**DOI:** 10.1186/s12877-020-01743-0

**Published:** 2020-09-07

**Authors:** Shuko Takahashi, Kozo Tanno, Yuki Yonekura, Masaki Ohsawa, Toru Kuribayashi, Yasuhiro Ishibashi, Shinichi Omama, Fumitaka Tanaka, Ryohei Sasaki, Megumi Tsubota-Utsugi, Eri Takusari, Makoto Koshiyama, Toshiyuki Onoda, Kiyomi Sakata, Kazuyoshi Itai, Akira Okayama, on behalf of the Iwate KENCO study group

**Affiliations:** 1 Division of Medical Education, Iwate Medical University, Idaidori 1-1-1, Yahaba-Cho, Shiwa-gun, Iwate 028-3694 Japan; 2 Department of Health and Welfare, Iwate Prefecture, Morioka, Iwate Japan; 3 Takemi Program in International Health, Harvard T.H. Chan School of Public Health, Boston, MA USA; 4 Department of Hygiene and Preventive Medicine, Iwate Medical University, Shiwa-gun, Iwate Japan; 5 St. Luke’s International University, Tokyo, Japan; 6 Morioka Tsunagi Onsen Hospital, Morioka, Iwate Japan; 7 Faculty of Humanities and Social Sciences, Iwate University, Morioka, Iwate Japan; 8 Department of Neurology and Gerontology, Iwate Medical University, Shiwa-gun, Iwate Japan; 9 Department of Neurosurgery, Iwate Medical University, Shiwa-gun, Iwate Japan; 10 Division of Nephrology and Hypertension, Department of Internal Medicine, Iwate Medical University, Shiwa-gun, Iwate Japan; 11 Iwate Health Service Association, Morioka, Iwate, Japan; 12 Health Service Center, Iwate University, Morioka, Japan; 13 Department of Nutritional Sciences, Morioka University, Takizawa, Japan; 14 Research Institute of Strategy for Prevention, Tokyo, Japan

**Keywords:** Aged, Japan, Long-term care insurance, Subjective health, Functional disability, Self-rated health

## Abstract

**Background:**

Although previous large population studies showed elderly with poor self-rated health (SRH) to be at a high risk of functional disability in Western countries, there have been few studies in which the association between SRH and functional disability was investigated in Japanese community dwellers. The association between SRH and functional disability, defined as certification of the long-term care insurance (LTCI) system, in Japanese elderly community dwellers was examined in this study.

**Methods:**

A total of 10,690 individuals (39.5% men, mean age of 71.4 years) who were 65 years of age or more who did not have a history of cardiovascular disease or LTCI certification were followed in this prospective study for 10.5 years. SRH was classified into four categories: good, rather good, neither good nor poor, and poor. A Cox proportional-hazards model was used to determine the hazard ratios (HRs) for the incidence of functional disability among the SRH groups for each sex.

**Results:**

The number of individuals with functional disability was 3377. Men who rated poor for SRH scored significantly higher for functional disability (HR [95% confidence interval]: poor = 1.74 [1.42, 2.14]) while women who rated rather good, neither good nor poor, and poor scored significantly higher for functional disability (rather good =1.12 [1.00, 1.25], neither good nor poor = 1.29 [1.13, 1.48], poor = 1.92 [1.65, 2.24]: p for trend < 0.001 in both sexes).

**Conclusion:**

Self-rated health, therefore, might be a useful predictor of functional disability in elderly people.

## Background

It has been reported that poor self-rated health (SRH) is an indication of underlying physical and mental abnormalities that are often difficult to detect through other measures of health such as body weight and blood pressure [1]. Previous studies have shown that poor SRH is associated with mortality [2], morbidity (e.g., cardiovascular disease) [3, 4], cancer [5], musculoskeletal disease [6], psychiatric diseases [7], and dementia [8].

The proportions of elderly people over the age of 65 years in populations have recently been increasing [9]. The government and private sector will both need to deal with these situations by addressing new approaches to social security. Japan has achieved the highest longevity ratings in the world (81 years of age for men and 87 years of age for women) [10], and the proportion of people aged 65 years or older is estimated to be 35.6 million (28.1% of the population) [11]. By 2042, it is predicted that the number of elderly people in Japan will reach 38.9 million [12]. There is an ongoing issue about sustaining senior health using social security in order to balance the financial burdens of pension and health care expenditure [13].

In 2000, the Japanese government implemented a long-term care insurance (LTCI) system for elderly individuals [14]. In 2016, the number of individuals using this service was approximately 6.3 million, which was 2.9-times larger than the number of initial certificates in 2000 [15]. Since the number of candidates is increasing, there is an urgent need to take measures of prevention in order to aid disability and intervene for those at high risk for various diseases, which in turn would prevent an increase in the financial burden.

Various endpoints including activities of daily living (ADL), basic ADL, LTCI, and independent criteria were used in previous studies. The studies mentioned above had small numbers of subjects (subjects ranging from 165 to 737) [16–18] except for one study [19]. Furthermore, the follow-up periods of the surveys conducted in those studies were also relatively short. The follow-up period in one study was only 1 year [18], while the follow-up periods after the baseline surveys were 5 years in the other studies [16, 17, 19]. To the best of our knowledge, there has been no large-scale prospective study on the association between SRH and functional disability in elderly people using LTCI with a long follow-up period (10 years or more).

Previous large population studies in Western countries have shown that elderly people with poor SRH are at a high risk for functional disabilities [20–23]. Poor SRH has also been shown to be strongly associated with mortality and functional decline in Japanese elderly residents [16–19]. The results of those studies indicate that SRH can be used for predicting future functional disabilities in Japanese.

Therefore, the aim of this study was to determine whether poor SRH increases the risk of functional disabilities in elderly Japanese people utilizing the certification of LTCI for 10 years.

## Methods

The research plan was approved by the Ethics Committee of Iwate Medical University Institute Review Board #1 (approval no. H13–33).

### Study population

**Fig. 1:**
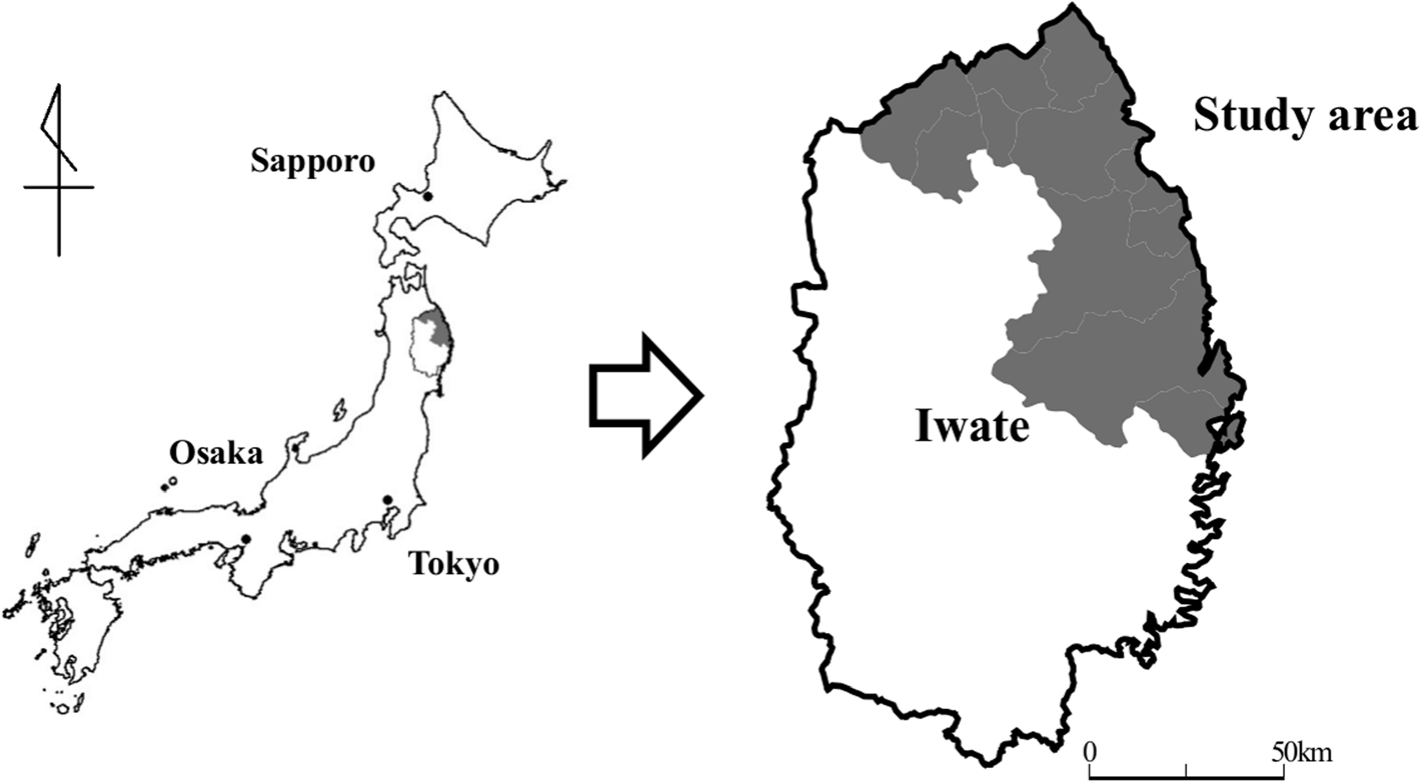
Map of the present study. The figure shows a map of Japan and a map of Iwate Prefecture. The gray areas shown the region of the study in northern Iwate (software used to create the map: Mandara for Windows Version 9.10. http://ktgis.net/mandara/index.php)

**Fig. 2:**
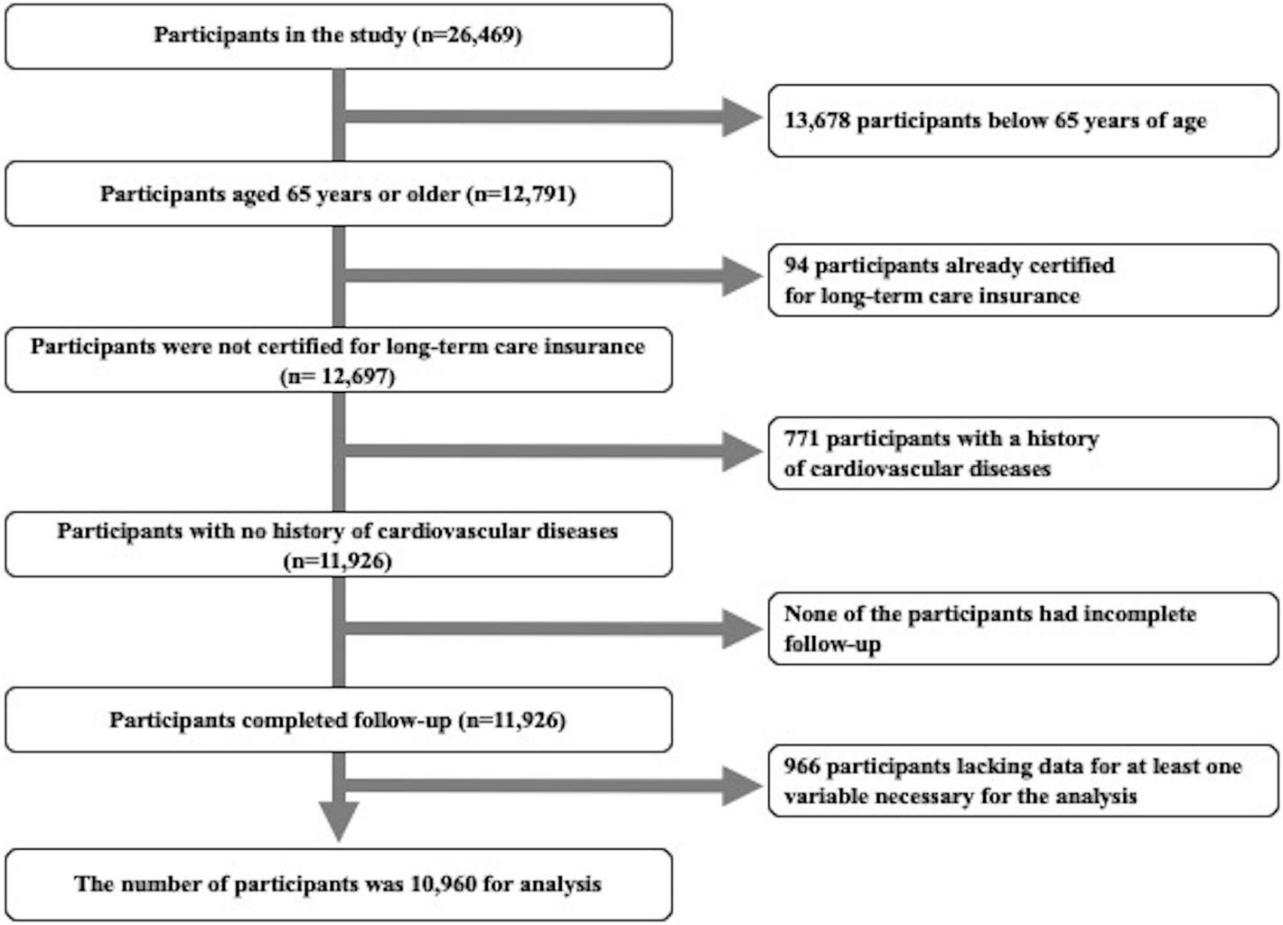
Flow chart showing selection of participants in the study. The original cohort consisted of 26,469 participants in the baseline survey. After exclusion of participants according to the exclusion criteria, a total of 10,960 participants remained

The original cohort of the Iwate-Kenpoku cohort (Iwate-KENCO) study, which is a population-based prospective study, consisted of residents Miyako, Ninohe, and Kuji districts in northern Iwate in Japan (Fig. 1). The Iwate-KENCO study began in 2002. The methodology has been described in detail in a previous report [24]. We performed the baseline survey from April to November in 2002 to 2004. Baseline examinations consisted of blood sampling, electrocardiography, anthropometrical examinations, blood pressure measurement, and a self-reported questionnaire with questions on self-rated health and medical history including the status of prescribed drugs. A total of 26,469 participants took part in multiphasic health checkups and agreed to complete the survey (acceptance rate, 84.5%). We longitudinally conducted follow-up of those participants using the dates of LTIC, which the city government recorded until December 31, 2014 (median follow-up period: 10.5 years). Patients (a) aged 65 years and older, (b) with no certification for LTCI of the time of the baseline survey, (c) with no history of cardiovascular diseases (stroke, heart failure, and myocardial infarction) at the time of the baseline survey, (d) with complete follow-up, and (e) without lacking data for at least one variable necessary for the analysis were included in the analysis. Data were analyzed for a total of 10,960 participants (4333 men and 6627 women, mean age of 71.4 years) (Fig. 2).

### Self-rated health

Self-rated health (SRH) was assessed by the single question: “How do you rate your health?” The five options included: “good,” “rather good,” “neither good nor poor,” “rather poor,” and “poor.” This assessment was then reclassified into four categories: “good,” “rather good,” “neither good nor poor,” and “poor”.

### Endpoint measurement

The endpoint of this study was incident functional disability, which is defined as receiving a new certification as an official recipient of the Japanese LTCI system [14]. It is nationally uniform criteria for LTCI, based on evaluation by the Certification Committee for long-term care need in municipalities. LTCI was used to validate physical and cognitive disabilities in elderly individuals in previous studies. One study showed that the levels of LTCI certification are closely associated with the ability to perform activities of daily living as assessed by the Barthel Index (Spearman’s coefficient = − 0.86) and with Mini-Mental State Examination scores (Spearman’s coefficient = − 0.42) [25]. Information on incident functional disability was obtained from the municipal public LTCI system database [26].

For the process of certification in the LTCI system, an elderly person or his caregiver (family or professional) contacts the municipal government to apply for care needs [27]. Trained local government officials conduct a home visit to evaluate the patient’s nursing care needs using a questionnaire regarding the patient’s current physical status, mental status, and medical procedures. The results are entered into a computer to calculate the applicant’s standardized scores for the seven dimensions of physical status and mental status, estimate the time required for the nine categories of care (grooming/bathing, eating, toileting, transferring, assistance with instrumental activities of daily living, behavioral problems, rehabilitation, and medical services), and assign a care-needs level based on the total estimated care minutes. The Nursing Care Needs Certification Board decides whether a LTCI certification will be provided or not after considering the results of the initial assessment using the primary care physician’s statement and notes written by the assessor during the home visit [28]. The levels of care consist of seven components: support levels 1 and 2 (i.e., individuals who require daily assistance) and care need levels 1 to 5 (i.e., individuals who are bedridden or in need of assistance such as assistance in eating and taking a bath). According to each level, applicants can receive the services covered by the fee for each service. Functional disability in any of the levels above LTCI items was defined as the endpoint [29].

### Baseline data and self-reported questionnaire

Anthropometrical examinations including measurements of body weight [kg] and height [cm] were performed. Body mass index was calculated as body weight [kg] divided by the square of height [m]. Blood pressure, anthropometric data, results of blood tests (serum levels of total cholesterol (TC; mg/dl), high-density lipoprotein cholesterol (HDLC; mg/dl), non-high-density lipoprotein cholesterol (non-HDLC; mg/dl), hemoglobin (Hb; g/dl), and glycosylated hemoglobin (HbA1c; %)), and estimated glomerular filtration rate (eGFR; mL/min/1.73 m^2^) were used for analysis [24, 29]. Participants answered a self-reported questionnaire that was prepared by the study committee. The questionnaire included questions about demographic characteristics (age and sex), socioeconomic status (job status, educational attainment, and marital status), health-related behaviors (smoking status, alcohol drinking status, exercise habits, and sleep duration), and past medical history (status of prescribed drugs for hypertension, diabetes mellitus, and dyslipidemia) [30]. Job status was dichotomized, having a job and non-employed or retired [30]. Education attainment was classified into 3 categories according to the duration of education: ≤ 9 years, 10–12 years and ≥ 13 years. Marital status was also dichotomized: single (unmarried, divorce or bereavement) or married. Smoking status (current, past smoker or non-smoker) and alcohol drinking status (never drinker, past drinker, drinker < 1 day per week, drinker 1–4 days per week, drinker ≥5 days per week) were also ascertained by the self-reported questionnaire. In women, alcohol drinking status was classified into 2 groups: never drinker and drinker. Regular exercise habits were classified into three categories; < 1 h per week (< 30 min 8 times per month), 1–2 h per week (30 to 59 min 8 times per month), and ≥ 2 h per week (≥ 60 min 8 times per month) [30]. Sleep duration was categorized into 3 groups: ≤ 6 h per day, 7–8 h per a day, ≥ 9 h per day. Clinical cardiovascular (CV) risk factors in three diseases (hypertension, diabetes mellitus and dyslipidemia) were assessed [29]. Hypertension was defined as systolic blood pressure of 140 mmHg or higher and/or diastolic blood pressure of 90 mmHg or higher, and/or the use of medications for a hypertensive state. Diabetes mellitus was defined as a non-fasting glucose concentration of 200 mg/dl or higher, and/or fasting blood glucose level of 126 mg/dl or higher, and/or HbA1_c_ value of 6.5% or higher, and/or the use of antidiabetic agents including insulin. Dyslipidemia was defined as serum TC level of 220 mg/dl or higher, serum HDLC level below 40 mg/dl, and/or the use of medications for hyperlipidemia. Interim CV diseases were also assessed utilizing a composite of stroke, myocardial infarction, and heart failure from the baseline study to the follow-up survey [31].

### Statistical analyses

All analyses were stratified by sex considering the difference in the baseline characteristics. Baseline factors are then compared among the four SRH groups. Continuous variables were expressed as means and standard deviation (SD), and categorical variables are expressed as proportions (%). Analysis of covariance (continuous variables) or the chi squared test (categorical variables) was utilized to examine the differences between variables. A linear trend test was also performed between the SRH categories and each risk factor.

Hazard ratios (HRs) of risk factors for future development of functional disability and their 95% confidential intervals (CIs) in the SRH groups (utilizing “good” SRH as the reference) were calculated using a multivariate Cox proportional hazard model after adjusting for covariates that were related to SRH in baseline characteristics of age, socioeconomic status, health-related behaviors, and objective indicators (i.e., BMI, systolic blood pressure, diastolic blood pressure, TC, non-HDLC, HDLC, Hb, HbA1c, and eGFR) and were used as confounding factors. A sequence of two models was run. In Model 1, we included the independent variables of SRH. Model 2 was further adjusted for variables with a significant relation to SRH in baseline characteristics. To evaluate the influence of the incidence of cardiovascular disease, time-dependent Cox regression analyses (adjusted for interim CV diseases) were calculated in the HRs of the incident of functional disability. To determine the additive effect of objective indicators, we compared the HRs for the risk of functional disability among the SRH groups with and without adjustment for objective indicators.

To avoid the possibility of participants already having developed sub-clinical serious diseases at the baseline survey and being certified for LTCI in the near future, similar analyses were performed excluding participants who were LTCI-certified within 2 years after the initial survey. To examine whether poor SRH contributes to the development of a more serious disability, Cox regression analyses were performed by using another LTCI category, thus, using two re-classified LTCI categories (i.e., LTCI care need level 2 or more and others). LTCI care need level 2 was defined as the requirement of assistance in at least one basic ADL task [32]. *P*-values < 0.05 were considered statistically significant. The Statistical Package for Social Sciences (SPSS) software program version 24.0 (IBM, Chicago, IL, USA) was used to perform statistical analyses.

## Results

The median follow-up period was 10.5 years (inter-quartile range, 10.1–12.4 years). During follow-up (112,490 person-years), 3377 participants were authorized as new recipients of LTCI for functional disability (1245 men and 2132 women, 3.3% per year). The median period to LTCI certification was 7.1 years (inter-quartile range, 4.3–9.3 years).

Baseline characteristics in the SRH groups are shown in Table 1. There was no significant difference in mean age among the SRH groups for both sexes. In men, SRH was worse in participants who were non-employed or retired, single, and never drinker and who had a low frequency of regular exercise, short sleep duration (≤ 6 h), long sleep duration (≥ 9 h), hypertension, and diabetes mellitus. Worse SRH was also related to higher levels of HbA1c and non-HDLC, lower levels of DBP and Hb and lower eGFR. In women, SRH was worse in participants who were having a job, participants who never smoked, and participants who had a low frequency of regular exercise, hypertension, and diabetes mellitus. Furthermore, worse SRH was related to a high level of HbA1c, low levels of TC and non-HDLC, and low eGFR.

**Table 1:** Characteristics of participants in the self-rated health groups (*n* = 10,960)

**Men (*n*** **= 4333)**		**Good (*n*** **= 1412)**	**Rather good (*n*** **= 1990)**	**Neither good nor poor (*n*** **= 635)**	**Poor (*n*** **= 296)**	***P*** **for trend**
**Age**	**Age (yr)**	71.7 (5.0)	71.7 (4.8)	71.6 (4.8)	72.6 (5.0)	0.307
**Anthropometrical examinations**	**BMI (kg/m**^**2**^**)**	23.6 (2.8)	23.7 (2.9)	23.6 (3.1)	23.6 (3.2)	0.901
**Blood pressure**	**SBP (mmHg)**	134.8 (20.4)	133.3 (19.0)	134.3 (20.9)	132.0 (19.2)	0.062
	**DBP (mmHg)**	78.3 (10.5)	77.7 (10.4)	78.1 (11.0)	76.4 (10.2)	0.022
**Biochemical data**	**TC (mg/dl)**	189.1 (31.0)	189.7 (30.9)	185.6 (31.9)	188.0 (32.4)	0.096
	**HDLC (mg/dl)**	56.2 (15.4)	56.0 (15.2)	56.3 (14.8)	54.3 (15.1)	0.197
	**Non-HDLC (mg/dl)**	132.9 (31.0)	133.7 (30.7)	129.4 (31.8)	133.7 (31.7)	0.022
	**Hb (g/dl)**	14.5 (1.3)	14.4 (1.3)	14.4 (1.3)	14.2 (1.5)	0.014
	**HbA1c (%)**	5.5 (0.7)	5.6 (0.8)	5.6 (0.8)	5.7 (0.9)	< 0.001
	**eGFR (mL/min/1.73m**^**2**^**)**	71.3 (8.7)	70.5 (9.1)	70.3 (9.9)	69.0 (11.4)	< 0.001
**Job status**	**Non-employed or retired**	57.5	61.7	66.9	67.9	< 0.001
**Educational attainment**	**≤ 9 yrs**	72.7	68.8	68.7	68.2	0.097
	**10–12 yrs**	19.3	22.8	22.8	23.6	
	**≥13 yrs**	8.1	8.4	8.5	8.1	
**Marital status**	**Single**	8.6	9.9	11.7	12.8	0.005
**Smoking status**	**Never smoker**	45.8	40.5	39.7	36.1	0.414
	**Past smoker**	29.2	36.2	36.1	44.3	
	**Current smoker**	25.0	23.3	24.3	19.6	
**Drinking status**	**Never drinker**	28.3	26.1	22.7	32.4	< 0.001
	**Past drinker**	7.6	10.1	14.6	21.3	
	**Drinker < 1 day per week**	5.9	7.5	8.0	7.1	
	**Drinker 1–4 days per week**	16.3	14.5	13.1	11.8	
	**Drinker ≥ 5 days per week**	41.9	41.8	41.6	27.4	
**Exercise habits**	**< 1 h per week**	70.2	69.9	73.4	79.7	0.006
	**1–2 h per week**	8.1	7.2	6.1	5.4	
	**> 2 h per week**	21.7	22.9	20.5	14.9	
**Sleep duration**	**≤ 6 h**	8.4	8.8	7.7	9.5	0.049
	**7–8 h**	54.8	52.6	49.4	48.6	
	**≥ 9 h**	36.8	38.5	42.8	41.9	
**Hypertension**	**Hypertension**	51.9	55.7	58.7	57.1	0.005
**Diabetes mellitus**	**Diabetes mellitus**	8.1	11.9	13.5	17.6	< 0.001
**Dyslipidemia**	**Dyslipidemia**	27.3	29.4	24.6	35.8	0.164
**Interim CV diseases**	**Interim CV diseases**	12.7	11.3	11.5	12.2	0.510
**Women (*n*** **= 6627)**		**Good (*n*** **= 1624)**	**Rather good (*n*** **= 3292)**	**Neither good nor poor (*n*** **= 1143)**	**Poor (*n*** **= 568)**	***P*** **for trend**
**Age**	**Age (yr)**	71.1 (4.6)	71.1 (4.5)	71.3 (70.9)	71.4 (4.5)	0.638
**Anthropometrical examinations**	**BMI (kg/m**^**2**^**)**	24.3 (3.3)	24.2 (3.4)	24.5 (3.6)	24.5 (3.8)	0.084
**Blood pressure**	**SBP (mmHg)**	130.3 (19.4)	131.7 (19.7)	132.2 (20.2)	129.9 (18.7)	0.450
	**DBP (mmHg)**	74.6 (10.4)	75.2 (10.4)	75.2 (10.6)	74.1 (10.4)	0.053
**Biochemical data**	**TC (mg/dl)**	209.4 (31.2)	206.9 (30.4)	206.5 (30.8)	205.7 (30.9)	0.004
	**HDLC (mg/dl)**	59.8 (14.2)	59.8 (14.6)	59.7 (14.1)	59.6 (14.1)	0.786
	**Non-HDLC (mg/dl)**	149.7 (31.3)	147.1 (29.8)	146.8 (30.5)	146.1 (30.5)	0.015
	**Hb (g/dl)**	13.0 (1.1)	13.0 (1.1)	12.9 (1.1)	13.0 (1.1)	0.249
	**HbA1c (%)**	5.6 (0.6)	5.6 (0.6)	5.6 (0.7)	5.7 (0.8)	< 0.001
	**eGFR (mL/min/1.73m**^**2**^**)**	71.9 (8.5)	71.9 (8.1)	71.4 (9.0)	70.4 (9.7)	0.001
**Job status**	**Non-employed or retired**	28.6	27.0	22.0	19.2	< 0.001
**Educational attainment**	**≤ 9 yrs**	79.1	77.7	79.5	82.0	0.139
	**10–12 yrs**	17.5	17.5	17.3	15.3	
	**≥13 yrs**	3.3	4.7	3.1	2.6	
**Marital status**	**Single**	36.5	36.1	38.6	40.5	0.054
**Smoking status**	**Never smoker**	98.3	98.9	99.0	99.6	0.008
	**Past smoker**	0.7	0.4	0.4	0.4	
	**Current smoker**	0.9	0.7	0.5	0.0	
**Drinking status**	**Never drinker**	88.2	89.5	89.1	88.2	0.537
	**Past drinker**	0.5	0.6	1.1	2.1	
	**Drinker < 1 day per week**	4.2	3.5	3.5	2.5	
	**Drinker 1–4 days per week**	4.7	4.4	4.5	4.8	
	**Drinker ≥ 5 days per week**	2.3	2.0	1.8	2.5	
**Exercise habits**	**< 1 h per week**	78.4	81.8	83.0	85.2	< 0.001
	**1–2 h per week**	8.0	7.4	7.8	8.1	
	**> 2 h per week**	13.6	10.7	9.2	6.7	
**Sleep duration**	**≤ 6 h**	12.1	12.5	13.6	17.6	0.552
	**7–8 h**	61.9	61.1	56.4	50.9	
	**≥ 9 h**	25.9	26.5	29.9	31.5	
**Hypertension**	**Hypertension**	48.3	55.8	57.8	60.6	< 0.001
**Diabetes mellitus**	**Diabetes mellitus**	6.0	6.6	8.7	11.3	< 0.001
**Dyslipidemia**	**Dyslipidemia**	43.2	42.5	42.3	46.3	0.449
**Interim CV diseases**	**Interim CV diseases**	6.5	7.4	7.5	6.0	0.888

Abbreviations: *BMI* body mass index, *CV diseases* cardiovascular disease, *DBP* diastolic blood pressure, *eGFR* estimated glomerular filtration rate, *Hb* hemoglobin, *HbA1c* glycosylated hemoglobin, *HDLC* high-density lipoprotein cholesterol, *Non-HDLC* non-high-density lipoprotein cholesterol, *SBP* systolic blood pressure, *TC* total cholesterol

Continuous variables were expressed as the mean (standard deviation) and Categorical variables were expressed as the proportion

The difference between the variables and the trend were tested by using an analysis of covariance test (continuous variables) or the chi-squared test (categorical variables)

**Fig. 3:**
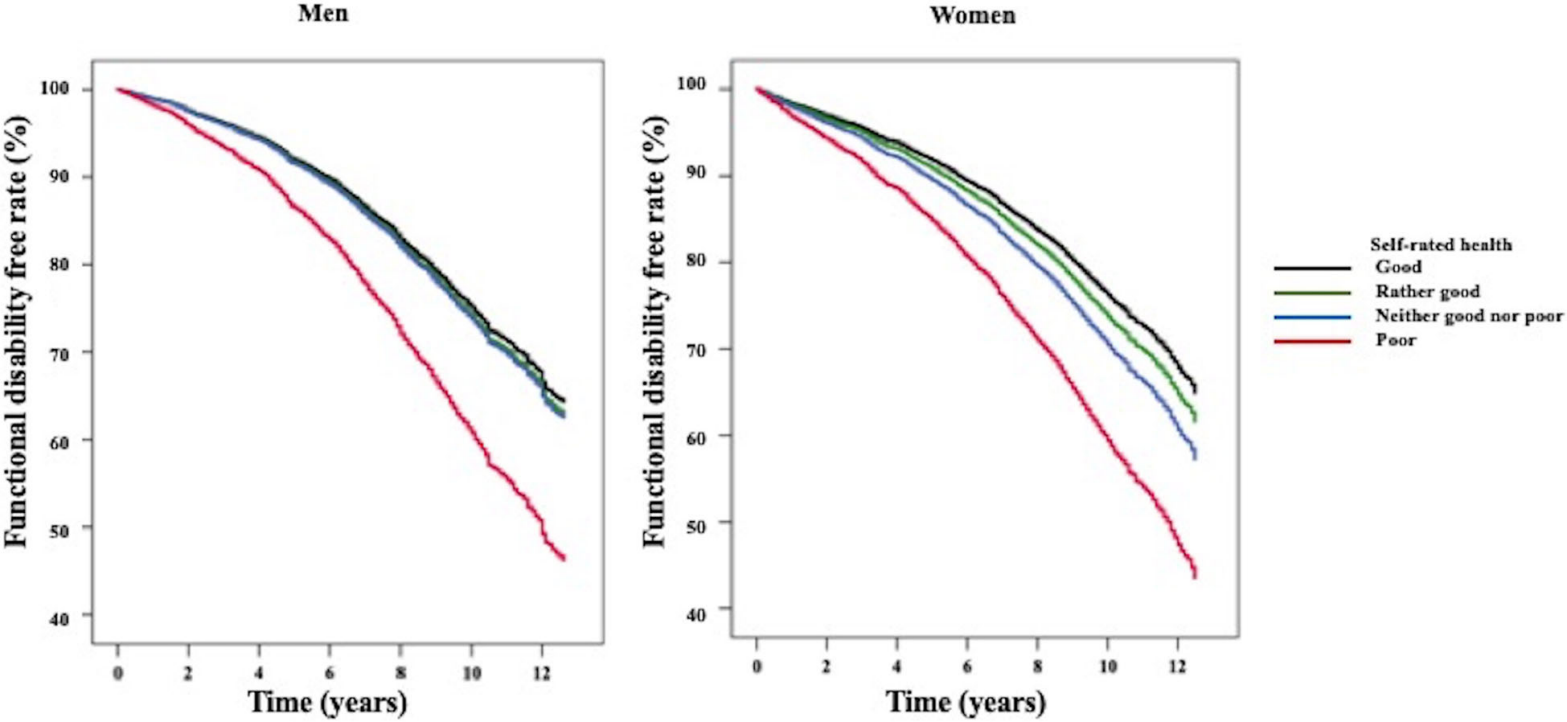
The cumulative survival curve of functional disability-free rates in the self-rated health groups

Table 2 shows the hazard ratios of functional disability among the SRH groups. The percentages of participants with functional disability increased with worsening of SRH (percentage in men: good SRH group, 25.9%; rather good SRH group, 28.4%; neither good nor poor SRH group, 29.6%; poor SRH group, 42.2%; percentages in women: good SRH group, 27.6%; rather good SRH group, 30.7%; neither good nor poor SRH group, 35.1%; poor SRH group, 47.7%). HRs of functional disability were found to be significantly higher in the neither good nor poor SRH group, and poor SRH group for men (HR [95% CI] in men: neither good nor poor SRH group = 1.21 [1.02, 1.45], poor SRH group = 2.07 [1.69, 2.54]) (Additional file 1). In women, HRs were significantly higher in three SRH groups (HR [95% CI]: rather good SRH group = 1.15 [1.03, 1.28], neither good nor poor SRH group = 1.39 [1.21, 1.59], and poor SRH group = 2.13 [1.83, 2.48]). After adjustments for covariates were made, these differences disappeared in the neither good nor poor SRH group for men but remained statistically significant in the other groups in Model 2. There were significant linear trends between the SRH groups and functional disability for both sexes (*p* < 0.001). In men, HRs for functional disability were found to be significantly higher in participants who had a high HbA1c level, who were non-employed or retired, who had a single marital status, and who had a long sleep duration (≥ 9 h), while HRs for functional disability were found to be significantly lower in participants with a high Hb level and high eGFR and who were drinkers 1–4 days per week. In women, HRs for functional disability were found to be significantly higher in participants who had a high level of HbA1c, who were non-employed or retired, who had a single marital status and who had a low frequency of physical exercise (< 1 h per week), while HRs for functional disability were found to be significantly lower in participants with a high eGFR. Figure 3 showes the cumulative survival curve of functional disability-free rates in the SRH groups. The differences in cumulative probability of functional disability-free rates among the SRH groups were significant (*p* < 0.001). Utilizing time-dependent Cox regression analysis with incorporation of the interim CV diseases, the HR for functional disability showed significant increases for men in the poor SRH group and for women in three SRH groups (Additional file 2).

**Table 2:** Hazard ratios for functional disability among the self-rated health groups utilizing a multivariate Cox regression analysis

	Self-rated health							***P*** for trend
	Good	Rather good		Neither good nor poor		Poor		
**Men (*n*** **= 4333)**								
**Number of participants**	1412	1990		635		296		
**Number of cases (person-years/1000 person-years)**	366 (2.55)	566 (3.87)		188 (1.21)		125 (0.71)		
**HR, 95% CI**	1 (reference)	1.04	0.92, 1.19	1.06	0.89, 1.27	1.74	1.42, 2.14	< 0.001
**Women (*n*** **= 6627)**								
**Number of participants**	1624	3292		1143		568		
**Number of cases (person-years/1000 person-years)**	449 (3.14)	1011 (6.92)		401 (2.58)		271 (1.55)		
**HR, 95% CI**	1 (reference)	1.12	1.00, 1.25	1.29	1.13, 1.48	1.92	1.65, 2.24	< 0.001

Abbreviations: *CI* confidence interval, *HR* hazard ratios

Cox regression analysis adjusted for the variables below

Men: diastolic blood pressure, non-high-density lipoprotein cholesterol, hemoglobin, glycosylated hemoglobin, estimated glomerular filtration rate, job status, marital status, alcohol drinking status, regular exercise habits, and sleep duration

Women: total cholesterol, non-high-density lipoprotein cholesterol, glycosylated hemoglobin, estimated glomerular filtration rate, job status, marital status, and regular exercise habits

The HRs for the risk of functional disability were further compared in the SRH groups with and without adjustments for objective indicators (Additional file 3). The HRs for the risk of functional disability remained significantly high in the poor SRH group in both models. After excluding sub-clinical cases, a significant association was found only in the poor SRH group for men, but significant associations remained in all three SRH groups for women (Table 3). In analysis of severe functional disability, the HRs for men were attenuated in all three SRH groups and a significant association was found for the poor SRH group. The HRs for functional disability for women were attenuated, but significant associations remained in the worst group. The linear trends show significant associations for both sexes in these analyses.

**Table 3:** Hazard ratios for functional disability among the self-rated health groups excluding participants with sub-clinical and severe cases

	Self-rated health							***P*** for trend
	Good	Rather good		Neither good nor poor		Poor		
**Functional disability excluded participants who were sub-clinical cases**								
**Men (*n*** **= 4126)**								
**Number of participants**	1356	1899		605		266		
**Number of the cases (person-years/1000 person-years)**	342 (2.52)	517 (3.82)		172 (1.19)		102 (1.50)		
**HR, 95% CI**	1 (reference)	1.02	0.89, 1.17	1.05	0.87, 1.27	1.58	1.26, 1.98	0.001
**Women (*n*** **= 6323)**								
**Number of participants**	1569	3166		1081		507		
**Number of cases (person-years/1000 person-years)**	410 (3.10)	918 (6.83)		349 (2.52)		213 (1.50)		
**HR, 95% CI**	1 (reference)	1.12	1.00, 1.26	1.25	1.08, 1.44	1.71	1.45, 2.02	< 0.001
**Functional disability in severe cases**								
**Men (*n*** **= 4333)**								
**Number of participants**	1412	1990		635		296		
**Number of cases (person-years/1000 person-years)**	258 (1.82)	401 (2.72)		129 (0.85)		84 (0.48)		
**HR, 95% CI**	1 (reference)	1.03	0.85, 1.26	1.12	0.86, 1.46	1.71	1.26, 2.33	0.004
**Women (*n*** **= 6627)**								
**Number of participants**	1624	3292		1143		568		
**Number of cases (person-years/1000 person-years)**	254 (1.84)	510 (3.57)		211 (1.37)		139 (0.83)		
**HR, 95% CI**	1 (reference)	0.98	0.85, 1.14	1.17	0.97, 1.40	1.62	1.31, 1.99	< 0.001

Abbreviations: *CI* confidence interval, *HR* hazard ratios

Cox regression analysis adjusted for the variables below

Men: diastolic blood pressure, non-high-density lipoprotein cholesterol, hemoglobin, glycosylated hemoglobin, estimated glomerular filtration rate, job status, marital status, alcohol drinking status, regular exercise habits, and sleep duration

Women: total cholesterol, non-high-density lipoprotein cholesterol, glycosylated hemoglobin, estimated glomerular filtration rate, job status, marital status, and regular exercise habits

## Discussion

In the present study, SRH in the baseline survey was found a strong predictor of functional disability by utilizing certification of LTCI in a community of Japanese elderly dwellers. A linear relationship was found between the incidence of functional disability and SRH. In addition, poorer SRH group was significantly higher risk for functional disability in analysis excluding participants who were functionally disabled within the initial 2 years of the survey. Furthermore, the association between SRH and functional disability remained significant in analysis of the endpoint for severe cases of functional disability.

Most of the previous studies on the relationship between poor SRH and functional disability were conducted in Western countries [20–23, 33]. The results of our study are in line with the results of studies in Western countries. Several studies have shown a relationship between SRH and the development of functional disability in Japan. Haga et al. demonstrated an association between SRH and ADL that were rated on the basis of five items (e.g., walking and dressing) [34]. Hirosaki et al. revealed an association between SRH and functional ability that was evaluated on the basis of basic ADL using seven items in community-dwelling Japanese elderly adults [17]. Compared with our study, these studies had a smaller sample size (124 in Haga’s study, and 654 in Hirosaki’s study). Additionally, the endpoints in those studies (ADL and basic ADL) were different from the endpoint in this study although the focus in both of those studies had similarities with the focus of the current study in terms of elucidating the association of SRH with functional disability. In the present study, functional disability as the endpoint was defined according to the levels of the abovementioned LTCI items (from support levels 1 and 2 and care need levels 1 to 5). People in support level 1 were defined as “those with limited ability to perform instrumental activities of daily living but remaining independent in basic activities of daily living.” [25]. This endpoint is compatible with the endpoint of Lee’s study and Hirai’s study in which functional disability was defined as disability including IADL limitations. Lee investigated the extent to which elderly people’s self-assessments of general health including SRH predicted functional decline in the US Longitudinal Study of Aging (LSOA) [21]. The odds ratios of functional decline were significantly higher in those who responded good, fair, or poor for SRH than in those who responded excellent (odds ratios [95% CI]: good SRH group = 1.43 [1.16, 1.76] and fair/poor SRH group = 1.56 [1.20, 2.03]). Although analysis with stratification by sex was not performed in Lee’s study, the classification of SRH (which was divided into four categories) was compatible with that in our study, and the magnitude of risk for functional disability was similar to that of our results. Hirai et al. examined risk factors for certification of LTCI in community dwelling elderly [19]. SRH was significantly related to increased risk of onset of LTCI in persons in support level 1 or more (HR [95% CI]: men in the poor SRH group = 2.79 [2.25, 3.47]; women, in the poor SRH group = 2.28 [1.91, 2.73]). Although the endpoint in Hirai’s study was exactly the same as that in our study, the classification of SRH (which was divided into two categories [good vs. poor]) was not compatible with that in our study. Furthermore, the HRs of risk for functional disability were higher in Hirai’s study than in our study possibly due to dichotomizing rather poor and poor as a poor SRH group. Our results suggest that poor SRH could be a predictor of functional disability even with some IADL limitations. Since SRH has also been recommended as a tool for disease risk screening [1], our results will be important role for estimation of the risk of functional disability in a general population.

The strength of this study was that we showed an association between SRH and functional disability in a large-scale prospective study in elderly community dwellers in East Asia over 10 years. Additional analyses such as analysis with exclusion of early cases and severe cases in endpoints improved the validity of our study. In addition, we showed an between SRH and functional disability even when adjustments were made for association objective indicators.

The results obtained by using the two models indicated that the association between SRH and functional disability was explained to some extent by confounding factors such as clinical indicators, social factors, and lifestyle, but there might have been residual confounders such as household income and the existence of mental disorders that were not incorporated in the models. Unfavorable social risk factors (non-employed or retired and single marital status) and unhealthy objective indicators (low Hb level, low eGFR, and high HbA1c level) contributed to the higher risk of functional disability. These results are consistent with the results of previous studies [35–37]. People with unfavorable social status and unhealthy status might conceive their health as being poor, which consequently lead to be functional disability.

CV diseases are well-known risks for future functional disability [38–41]. The main reasons for receiving LTCI were cerebrovascular disease (stroke) (21.5%) followed by dementia (15.3%) [42]. Several studies have demonstrated that poor SRH is a consistent predictor of the incidence of several type of CV diseases such as stroke, coronary artery disease, and heart failure [4, 43, 44]. Based on these facts, we initially hypothesized that poor SRH related to functional disability was mediated by physical dysfunctions caused by CV diseases. However, HRs of the incident risk for functional disability did not change in time-dependent Cox regression analyses adjusted for interim CV diseases. Another possibility for the higher incidence of functional disability in people with poor SRH is the influence of dementia. Poor SRH was shown to be a risk for incident dementia [8], and decline in cognitive function was shown to be associated with LTCI after an 18-month observation using a cognitive performance scale [45]. Lin et al. reported that people with dementia who lived alone had a higher risk of care needs than did those without dementia [46]. Although we were not able to evaluate the causes of functional disability due to a lack of information about cognitive dysfunction, we speculate that dementia relates to functional disability in elderly adults with poor SRH.

Previous studies have shown that poor SRH is associated with objective indicators such as serum cholesterol and HbA1c levels [47, 48], and studies have shown that cardiovascular risk factors are associated with functional disability [49, 50]. In most previous studies, only subjective indicators such as self-reported information or interview-based information was examined even though these subjective items cannot be avoided by a recall bias [17–19]. Jylhä et al. mentioned the importance of investigating SRH adjusted for biological or clinical variables [51]. Therefore, adjustment for these clinical indicators gives light to new findings in the relationship between SRH and functional disability. In the analysis in the present study, after adjusting for clinically verified health indicators (such as results of anthropometrical examination and blood tests), poor SRH remained independently associated with functional disability. Even though there may still be residual confounding, e.g., inflammatory biomarkers, that does not allow for full adjustment, we could show a significant association between SRH and functional disability independent of objective indicators.

Though the mechanisms underlying the predictive ability of SRH for functional disability were not revealed in this study, there are some possible explanations of this pathway from poor SRH to functional disability. It is believed that the incidence of functional disability in the present study was affected by physical impairment and also cognitive dysfunction [42]. In pathophysiology, there is an association between SRH and subcortical dysfunction, which are often linked to vascular risk factors [52]. Gray matter atrophy and cortical thinning, as a consequence of microvascular diseases, cause neuronal damage [53]. Neuronal damage might lead to cognitive dysfunction by expressing social vulnerability [52]. In addition, recent studies have shown that SRH is related to several inflammatory biomarkers including C-reactive protein and cytokines [30, 54]. High IL-6 plasma levels are associated with functional impairment in elderly individuals with vascular dementia [55]. A previous study showed that albuminuria predicted future functional disability [29]. Given these findings, poor SRH might be an alternative index of functional disability with physical impairment and cognitive dysfunction caused by asymptomatic brain infarction or endothelial dysfunction.

### Limitations

The present study had several limitations. First, variables concerning psychological factors (e.g., depression) or subjective symptoms (which are associated with poor SRH and functional disability) were not included in the study [17]. The association of poor SRH with functional disability remained significant in a sub-group analysis (Additional file 4). Second, cancer incidence could not be included in the analysis due to a lack of information. Third, the actual causes of functional disability (e.g., physical and cognitive dysfunctions) were not identified. Notably, we could not incorporate the influence of dementia, which is one of the main reasons for receiving LTCI. Fourth, there might be reverse causation between poor SRH and functional disability. Because a significant association between SRH groups and participants with functional disability was found in the analysis excluding individuals with LTCI certification within 2 years from the initial survey, the influence of reverse causation might be small. Fifth, the generalizability of the results is not clear since the prevalence of LTCI was lower in the current study than in representative national surveys [56, 57]. Furthermore, since participants in this study received a health check-up, they might be healthier or have more awareness of health than non-participants. Although this assumption might be underestimated, it is believed that the relationship between SRH and functional disability shows a true association. Finally, the distribution of SRH was not similar to national representative data. We showed the distribution of SRH between the data of the Comprehensive Survey of Living Conditions in Japan in 2004 and the data of our study [58] (Additional file 5). In the data of the Comprehensive Survey of Living Conditions, neither good nor poor SRH group had a higher percentage of participants. In the present study, rather good SRH group had a higher percentage of participants for both sexes. Individuals in the present study tended to provide more favorable answers than did participants in the national survey. Our participants might be healthier citizens. Present results might not be generalizable to a broader sample of elderly individuals.

## Conclusion

Poorer SRH was significantly related to higher hazard ratios for future functional disability in both sexes among the Japanese community-dwelling elderly participants. Although it is difficult to accurately predict future functional disability using a single question, SRH might be useful for predicting later functional disability in elderly people as a simple, low-cost questionnaire, thus allowing for identification of high-risk individuals for future functional disability at an early stage.

## Supplementary information

**Additional file 1.** Hazard ratios for functional disability in the self-rated health groups using multivariate Cox regression analysis with exclusion of variables not related to self-rated health in the baseline comparison.

**Additional file 2.** Time-dependent Cox regression analysis to predict functional disability in the self-rated health groups.

**Additional file 3 **Comparison of hazard ratios for the risk of functional disability in the self-rated health groups with and without *objective indicators (adjusted model)*.

**Additional file 4.** Hazard ratios for future functional disability in the self-rated health groups stratified by baseline data utilizing multivariate Cox regression analysis.

**Additional file 5.** Comparison of the distribution of self-rated health between Comprehensive Survey of Living Conditions in Japan in 2004 and the present study.

## Data Availability

The data that support the findings of this study are available from KENCO study group (Iwate Medical University) but restrictions apply to the availability of these data, which were used under license for the current study, and so are not publicly available. Data are however available from the authors upon reasonable request and with permission of KENCO study group (Iwate Medical University).

## Abbreviations

ADL: Activities of daily living
BMI: Body mass index
CI: Confidence interval
CV diseases: Cardiovascular diseases
DBP: Diastolic blood pressure
eGFR: Estimated glomerular filtration rate
Hb: Hemoglobin
HbA1c: Glycosylated hemoglobin
HDLC: High-density lipoprotein cholesterol
HR: Hazard ratio
LTCI: Long-term care insurance
non-HDLC: Non-high-density lipoprotein cholesterol
SBP: Systolic blood pressure
SD: Standard deviation
SRH: Self-rated health
TC: Total cholesterol
