# Supplementary material for: Poor self-rated health predicts the incidence of functional disability in elderly community dwellers in Japan: a prospective cohort study
Source: BMC Geriatr. 2020 Sep 7;20:328. doi: 10.1186/s12877-020-01743-0 (PMC7487733; doi:10.1186/s12877-020-01743-0)
Supplement: Supplementary file 2 — Additional file 2. Time-dependent Cox regression analysis to predict functional disability in the self-rated health groups. [file 12877_2020_1743_MOESM2_ESM.pdf]

Additional File 2. Time-dependent Cox regression analysis to predict functional disability in the self-rated health groups

		Men			Women		
		HR	95% CI	*P* values	HR	95% CI	*P* values
**Blood pressure**	**DBP (mmHg)**	1.00	0.99, 1.00	0.661			
**Biochemical data**	**TC (mg/dl)**				1.00	0.99, 1.00	0.063
	**Non-HDLC (mg/dl)**	1.00	1.00, 1.00	0.083	1.00	1.00, 1.00	0.947
	**Hb (g/dl)**	0.94	0.90, 0.98	0.002			
	**HbA1c (%)**	1.13	1.05, 1.22	0.001	1.13	1.07, 1.20	<0.001
	**eGFR (mL/min/1.73m^2)**	0.98	0.97, 0.98	<0.001	0.97	0.96, 0.97	<0.001
**Job status**	**Non-employed or retired**	1.39	1.23, 1.57	<0.001	1.38	1.24, 1.53	<0.001
**Marital status**	**Single (ref: married)**	1.79	1.53, 2.11	<0.001	1.39	1.28, 1.52	<0.001
**Drinking status**	**Past drinker (ref: Never drinker)**	1.00	0.83, 1.21	0.998			
	**Drinker < 1 day per week**	0.91	0.72, 1.15	0.405			
	**Drinker 1-4 days per week**	0.73	0.60, 0.88	0.001			
	**Drinker ≥5 days per week**	1.01	0.88, 1.16	0.930			
**Exercise habits**	**<1h per week (ref: > 2h per week)**	1.16	1.00, 1.34	0.044	1.17	1.01, 1.35	0.031
	**1-2 h per week**	1.08	0.85, 1.38	0.525	0.94	0.76, 1.17	0.598
**Sleep duration**	**≤ 6 h (ref: 7-8 h)**	0.95	0.77, 1.18	0.633			
	**≥ 9 h**	1.33	1.18, 1.50	<0.001			
**Self-rated health**	**Rather good (ref: good)**	1.05	0.92, 1.20	0.496	1.13	1.01, 1.26	0.037
	**Neither good nor poor**	1.06	0.89, 1.26	0.539	1.29	1.13, 1.48	<0.001
	**Poor**	1.76	1.43, 2.16	<0.001	1.93	1.66, 2.24	<0.001

Abbreviations: CI, confidence interval; DBP, diastolic blood pressure; eGFR, estimated glomerular filtration rate; Hb, hemoglobin; HbA1c, glycosylated hemoglobin; HR, hazard ratios; non-HDLC, non-high-density lipoprotein cholesterol; TC, total cholesterol

Time dependent Cox regression analysis adjusted for the variables below

Men: diastolic blood pressure, non-high-density lipoprotein cholesterol, hemoglobin, glycosylated hemoglobin, estimated glomerular filtration rate, job status, marital status, alcohol drinking status, regular exercise habits, and sleep duration

Women: total cholesterol, non-high-density lipoprotein cholesterol, glycosylated hemoglobin, estimated glomerular filtration rate, job status, marital status, and regular exercise habits
