# Supplementary material for: Poor self-rated health predicts the incidence of functional disability in elderly community dwellers in Japan: a prospective cohort study
Source: BMC Geriatr. 2020 Sep 7;20:328. doi: 10.1186/s12877-020-01743-0 (PMC7487733; doi:10.1186/s12877-020-01743-0)
Supplement: Supplementary file 3 — Additional file 3 Comparison of hazard ratios for the risk of functional disability in the self-rated health groups with and without objective indicators (adjusted model). [file 12877_2020_1743_MOESM3_ESM.pdf]

Additional File 3. Comparison of hazard ratios for the risk of functional disability in the self-rated health groups with and without objective indicators (adjusted model)

		Men (n=4333)						Women(n=6627)					
		Without objective indicators			With objective indicators			Without objective indicators			With objective indicators		
		HR	95% CI	*P* values	HR	95% CI	*P* values	HR	95% CI	*P* values	HR	95% CI	*P* values
Blood pressure	DBP (mmHg)				1.00	0.99, 1.00	0.600						
Biochemical data	TC (mg/dl)										1.00	0.99, 1.00	0.063
	Non-HDLC (mg/dl)				1.00	1.00, 1.00	0.054				1.00	1.00, 1.00	0.939
	Hb (g/dl)				0.94	0.90, 0.98	0.003						
	HbA1c (%)				1.12	1.04, 1.21	0.003				1.13	1.07, 1.20	<0.001
	eGFR (mL/min/1.73m^2)				0.98	0.97, 0.98	0.757				0.97	0.96, 0.97	<0.001
Job status	Non-employed or retired	1.45	1.28, 1.64	<0.001	1.38	1.22, 1.56	0.012	1.48	1.34, 1.65	<0.001	1.38	1.24, 1.54	<0.001
Marital status	Single (ref: married)	1.81	1.54, 2.12	<0.001	1.79	1.52, 2.10	<0.001	1.46	1.34, 1.59	<0.001	1.39	1.28, 1.52	<0.001
Drinking status	Past drinker (ref: Never drinker)	1.06	0.87, 1.28	0.581	1.01	0.83, 1.23	0.910						
	Drinker < 1 day per week	0.91	0.72, 1.15	0.417	0.92	0.72, 1.16	0.459						
	Drinker 1-4 days per week	0.71	0.59, 0.87	0.001	0.73	0.60, 0.89	0.002						
	Drinker ≥5 days per week	0.93	0.81, 1.06	0.276	1.00	0.87, 1.15	0.955						

**Exercise habits**	**<1h per week (ref: > 2h per week)**	1.12	0.98, 1.30	0.107	1.14	0.99, 1.32	0.072	1.20	1.04, 1.39	0.013	1.18	1.02, 1.36	0.026
	**1-2 h per week**	1.07	0.84, 1.37	0.565	1.07	0.84, 1.36	0.601	0.97	0.79, 1.21	0.81	0.96	0.77, 1.19	0.69
**Sleep duration**	**≤ 6 h (ref: 7-8 h)**	0.96	0.77, 1.19	0.714	0.94	0.76, 1.17	0.596				1.06	0.93, 1.21	0.376
	**≥ 9 h**	1.35	1.20, 1.52	<0.001	1.33	1.18, 1.50	<0.001				1.14	1.04, 1.26	0.006
**Self-rated health**	**Rather good (ref: good)**	1.07	0.94, 1.22	0.322	1.04	0.92, 1.19	0.520	1.14	1.02, 1.28	0.020	1.12	1.00, 1.25	0.044
	**Neither good nor poor**	1.11	0.93, 1.32	0.271	1.06	0.89, 1.27	0.497	1.33	1.17, 1.53	<0.001	1.29	1.13, 1.48	<0.001
	**Poor**	1.84	1.50, 2.26	<0.001	1.74	1.42, 2.14	<0.001	2.05	1.76, 2.38	<0.001	1.92	1.65, 2.24	<0.001

Abbreviations: CI, confidence interval; DBP, diastolic blood pressure; eGFR, estimated glomerular filtration rate; Hb, hemoglobin; HbA1c, glycosylated hemoglobin; HR, hazard ratios; non-HDLC, non-high-density lipoprotein cholesterol; TC, total cholesterol
