# Supplementary material for: Poor self-rated health predicts the incidence of functional disability in elderly community dwellers in Japan: a prospective cohort study
Source: BMC Geriatr. 2020 Sep 7;20:328. doi: 10.1186/s12877-020-01743-0 (PMC7487733; doi:10.1186/s12877-020-01743-0)
Supplement: Supplementary file 4 — Additional file 4. Hazard ratios for future functional disability in the self-rated health groups stratified by baseline data utilizing multivariate Cox regression analysis. [file 12877_2020_1743_MOESM4_ESM.pdf]

Additional File 4. Hazard ratios for future functional disability in the self-rated health groups stratified by baseline data utilizing multivariate Cox regression analysis

			Men			Women		
			HR	95% CI	*P* values	HR	95% CI	*P* values
**Age**	**Age <75 years**	**Rather good (ref: good)**	1.10	0.91, 1.32	0.326	1.19	1.03, 1.38	0.021
		**Neither good nor poor**	1.22	0.96, 1.54	0.109	1.37	1.14, 1.64	0.001
		**Poor**	1.91	1.44, 2.54	<0.001	2.14	1.75, 2.61	<0.001
	**Age ≥75 years**	**Rather good (ref: good)**	1.09	0.90, 1.32	0.384	1.07	0.91, 1.27	0.404
		**Neither good nor poor**	1.10	0.84, 1.44	0.489	1.35	1.10, 1.67	0.004
		**Poor**	1.84	1.36, 2.49	<0.001	1.87	1.48, 2.37	<0.001
**Hypertension**	**Hypertension**	**Rather good (ref: good)**	0.98	0.82, 1.16	0.805	1.09	0.94, 1.26	0.263
		**Neither good nor poor**	0.96	0.76, 1.21	0.722	1.25	1.05, 1.49	0.012
		**Poor**	1.72	1.31, 2.24	<0.001	1.74	1.43, 2.11	<0.001
	**No hypertension**	**Rather good (ref: good)**	1.12	0.91, 1.37	0.286	1.09	0.92, 1.30	0.313
		**Neither good nor poor**	1.20	0.90, 1.58	0.217	1.26	1.01, 1.57	0.039
		**Poor**	1.67	1.20, 2.32	0.002	2.13	1.67, 2.72	<0.001
**Diabetes mellitus**	**Diabetes mellitus**	**Rather good (ref: good)**	1.08	0.71, 1.66	0.715	1.26	0.85, 1.86	0.256
		**Neither good nor poor**	1.17	0.69, 2.00	0.564	0.99	0.62, 1.58	0.966
		**Poor**	1.97	1.13, 3.44	0.017	1.89	1.18, 3.01	0.008
	**No diabetes mellitus**	**Rather good (ref: good)**	1.04	0.91, 1.20	0.548	1.11	0.99, 1.25	0.071
		**Neither good nor poor**	1.08	0.89, 1.30	0.456	1.33	1.15, 1.53	<0.001
		**Poor**	1.72	1.37, 2.16	<0.001	1.94	1.65, 2.28	<0.001
**Dyslipidemia**	**Dyslipidemia**	**Rather good (ref: good)**	1.21	0.94, 1.55	0.146	1.13	0.95, 1.34	0.158
		**Neither good nor poor**	1.09	0.76, 1.56	0.657	1.28	1.04, 1.57	0.021
		**Poor**	1.64	1.13, 2.39	0.010	2.02	1.61, 2.53	<0.001
	**No Dyslipidemia**	**Rather good (ref: good)**	0.99	0.85, 1.16	0.884	1.12	0.97, 1.30	0.128
		**Neither good nor poor**	1.04	0.85, 1.28	0.709	1.31	1.10, 1.56	0.003

**Poor**	1.85	1.44, 2.37	<0.001	1.89	1.54, 2.33	<0.001

Abbreviations: CI, confidence interval; HR, hazard ratios

Hazard ratios adjusted for the variables below

Men: diastolic blood pressure, non-high-density lipoprotein cholesterol, hemoglobin, glycosylated hemoglobin, estimated glomerular filtration rate, job status, marital status, alcohol drinking status, regular exercise habits, and sleep duration

Women: total cholesterol, non-high-density lipoprotein cholesterol, glycosylated hemoglobin, estimated glomerular filtration rate, job status, marital status, and regular exercise habits
