# Supplementary material for: Poor self-rated health predicts the incidence of functional disability in elderly community dwellers in Japan: a prospective cohort study
Source: BMC Geriatr. 2020 Sep 7;20:328. doi: 10.1186/s12877-020-01743-0 (PMC7487733; doi:10.1186/s12877-020-01743-0)
Supplement: Supplementary file 5 — Additional file 5. Comparison of the distribution of self-rated health between Comprehensive Survey of Living Conditions in Japan in 2004 and the present study. [file 12877_2020_1743_MOESM5_ESM.pdf]

Additional File 5. Comparison of the distribution of self-rated health between Comprehensive Survey of Living Conditions in Japan in 2004 and the present study

	Men		Women	
	Comprehensive Survey of Living Conditions	Present study	Comprehensive Survey of Living Conditions	Present study
**Self-rated health**	**%**	**%**	**%**	**%**
**Good**	16.0	32.6	11.5	37.5
**Rather good**	15.5	45.9	14.5	76.0
**Neither good nor poor**	44.6	14.7	45.6	26.4
**Poor**	23.9	6.8	26.7	13.1
